# Distribution and Evolution of the Bacteriophage WO and Its Antagonism With *Wolbachia*

**DOI:** 10.3389/fmicb.2020.595629

**Published:** 2020-11-13

**Authors:** Yun-heng Miao, Jin-hua Xiao, Da-wei Huang

**Affiliations:** ^1^Key Laboratory of Zoological Systematics and Evolution, Institute of Zoology, Chinese Academy of Sciences, Beijing, China; ^2^College of Life Sciences, Nankai University, Tianjin, China

**Keywords:** bacteriophage WO, *Wolbachia*, genomic structure, distribution pattern, coevolutionary pattern

## Abstract

The symbiosis system comprising eukaryotic hosts, intracellular bacterium *Wolbachia*, and temperate bacteriophages WO is widely spread through nearly half the number of arthropod species. The relationships between the three components of the system are extremely intricate. Even though the bacteriophage WO can have diverse influences on the ecology and evolution of *Wolbachia*, little is known about the distribution and evolution of the phages. To the best of our knowledge, this study is the first to report that in infected fig wasps (*Ceratosolen solmsi*, *Kradibia gibbosae*, and *Wiebesia pumilae*), the genomes of all the *Wolbachia* strains had only one cryptic WO prophage, which contained defects in the genomic structural modules. This phenomenon was contrary to the widely accepted understanding that *Wolbachia* with cryptic prophages usually possesses at least one intact WO prophage consisting of gene sequences of the head, baseplate, and tail modules, through which the prophage could form intact virions. In addition to the genetic structure features, the phylogenetic relationships of WO and *Wolbachia* also revealed that bacteriophage WO can horizontally spread among a certain genus or a group of insect hosts, nearly free from the restriction of the affiliation of *Wolbachia*. Combined with the vertical transmission along with *Wolbachia*, the wide spread of WO phages can be explained. Furthermore, the gender preference and functional module preference for transcriptional activity of the genes in cryptic WOs implied the antagonized coevolutionary pattern between WO prophages and their *Wolbachia* hosts.

## Introduction

*Wolbachia* is a genus of intracellular endosymbiotic bacteria belonging to the order Rickettsiale and is estimated to be distributed in more than 66% of arthropods and also shows mutualistic symbiosis in nematodes (Werren, 1997; Ferri et al., 2011). By phylogenetic analyses based on conserved coding genes used for multilocus sequence typing, such as 16S rRNAs and the *Wolbachia* surface protein (*wsp*) gene, *Wolbachia* has been classified into various strains under 18 supergroups (A–R) (Landmann, 2019). *Wolbachia* can manipulate the reproductive system of eukaryotic hosts using diverse methods, such as cytoplasmic incompatibility (CI), feminization of genetic male, parthenogenesis, and male killing (Werren et al., 2008), to avail the maternal transmission of its own population. It can also exert an influence on numerous processes in the host, including immune, behavioral, and metabolic processes (Landmann, 2019). Therefore, *Wolbachia* has great research potential as well as application value. For instance, it can be applied to control the populations of *Aedes albopictus* and *Aedes aegypti*, for inhibiting the widespread transmission of various arboviruses that are harmful to humans (Hoffmann et al., 2011; van den Hurk et al., 2012; Caragata et al., 2016).

WO, a bacteriophage that infects *Wolbachia*, is a λ phage-like temperate phage (Masui et al., 2001; Bordenstein et al., 2006). More than 80% of *Wolbachia* strains have WO-related gene fragments, and the infection proportion in supergroups A and B of *Wolbachia* is up to 90% (Gavotte et al., 2007). The CG contents and the codon preference of WO and *Wolbachia* bacteria are similar, so it has been speculated that WO and *Wolbachia* have coevolved for more than 100 million years (Masui et al., 2000). Therefore, there must be an intricate biological relationship between WO and *Wolbachia*.

As a temperate bacteriophage, WO has two different states. Viral DNA of the phage in the lysogenic state can be integrated into the genome of *Wolbachia* and duplicated with *Wolbachia*, which is usually called a prophage (Atanasoff, 1967). However, under certain conditions, the prophage can also convert into the lytic state, forming virions and causing the lysis of the bacteria (Echols, 1972). Prophages are under the selective pressure of their hosts, leading to various genomic defects of partial DNA, such as recombination, gene loss, or gradual degradation (Canchaya et al., 2003). The defective genomic prophages no longer have the ability to form virions and lyse host cells and are usually called defective prophages or cryptic prophages (Canchaya et al., 2003; Saridaki et al., 2011). Cryptic prophage WO is also found in the genome of *Wolbachia*. The most significant difference between intact and cryptic WO is that intact WO has a relatively complete gene module that codes for the proteins of the head, baseplate, and tail so that it can form active virions. Interestingly, *Wolbachia* with cryptic phages reported so far usually contain at least one intact prophage. However, there are exceptions. For example, in the *Wolbachia* strain of *w*Rec that infects the *Drosophila recens*, it was reported for the first time that the host contained only a WO prophage with a fragmented structure (Metcalf et al., 2014). It has also been reported that only one cryptic prophage WOSol was found in *Wolbachia* infecting the fig wasp *Ceratosolen solmsi*, and interestingly, despite being a cryptic prophage, more than half of the WOSol genes were still transcriptionally active (Wang et al., 2014), which was far more than the previously reported numbers of the active genes in cryptic prophage WORiB of *Drosophila simulans* in which only two *ank* genes and one methyltransferase gene (*met2*) were shown to have transcriptional activity (Biliske et al., 2011), indicating that cryptic phages WO could have multiple gene functions.

It has been known that bacteriophages can have diverse influences on the ecology and evolution of bacterial hosts. For example, bacteriophages provide beneficial genes to the bacterial host (Abedon and Lejeune, 2005; Wang et al., 2010) or mediate the horizontal transfer of genes (Wommack and Colwell, 2000). Similarly, the phage can also mediate horizontal transfer of *Wolbachia* bacterial genes (Wang G. H. et al., 2016). Recent researches have further revealed that phage WO possibly plays an important role in the induction of CI in insect hosts by *Wolbachia* bacteria (LePage et al., 2017; Lindsey et al., 2018; Shropshire et al., 2018). Nevertheless, our understanding of WO is still incomplete, and the cause for the wide distribution of the phage in *Wolbachia* strains is unknown. Even though some evidences have validated that WO can be transmitted horizontally across different *Wolbachia* strains (even belonging to different supergroups) coinfecting the same cell of the insect host (Bordenstein and Wernegreen, 2004; Kent et al., 2011), and the phylogeny of phage WO found in fig wasps has revealed the strong specificity for insect hosts (Wang N. et al., 2016), they are still not sufficient to explain the widespread existence of WO. Therefore, this study was carried out to understand the distribution and evolution of the phage.

In this study, based on the well-assembled genomes of the three *Wolbachia* strains, infecting three species of pollinating fig wasps (*C. solmsi*, *Kradibia gibbosae*, and *Wiebesia pumilae*) (Hymenoptera, Chalcidoidea), we discovered that each *Wolbachia* strain contained only one cryptic prophage WO without the presence of any intact WO. To the best of our knowledge, this is the first study to report multiple *Wolbachia* strains containing only one cryptic phage WO in a certain group of insect hosts. By combining the genomes of the three cryptic WO phages and the WO-related genomic data of other strains of *Wolbachia*, our studies provided evidence that could prove the horizontal transmission of the WO phage among a genus or a certain group of insect hosts, which can elucidate the wide spread of phage WO.

## Materials and Methods

### The Collection of Fig Wasp Samples

We collected the fig wasp samples for the three pollinating fig wasps species, with the species of *C. solmsi* from Dongguan, Guangdong province, China (N22°39′, E113°31′); *W. pumilae* from Huangshan, Anhui province, China (E118°33′, N29°72′); and *K. gibbosae* from Danzhou, Hainan province, China (N19°30′, E109°29′). All fig wasps were collected at the stage of adults and then identified and classified under microscope *Nikon SMZ80*. Each gender of every species was conserved into RNA hold for RNA extraction.

### Assembly of *Wolbachia* Genomes

The *Wolbachia* genomes were assembled based on the whole-genome sequencing data of the three fig wasp species in our laboratory (project accession number of PRJNA641212 for *W. pumilae* and *K. gibbosae*, PRJNA178998 for *C. solmsi*). We designed three different strategies to demonstrate the assembling procedures of each *Wolbachia* strain. (a) For the genome of *Wolbachia* infecting *C. solmsi* (*w*Csol), which was sequenced using second-generation Illumina HiSeq TM2000, the clean reads were aligned to the data set of the genomes of known *Wolbachia* using BLASTN (identity ≥ 70%, *e*-value ≤ 1e−5) to screen out homologous reads of *Wolbachia* for subsequent assembling using *SOAPdenovo2* (Luo et al., 2012). During the assembling process, the parameters were adjusted constantly until an optimal result was obtained. (b) For the genome of *Wolbachia* infecting *W. pumilae* (*w*Wpum), which was sequenced using third-generation *PacBio Sequel*, after assembling of corrected clean reads using *smartdenovo-1.0*, the assembly was polished using *pilon-1.22*. We then discovered an intact scaffold, which was highly homologous with *Wolbachia* (coverage ≥ 99.99%), and this scaffold was considered as the genome of *w*Wpum. (c) For the genome of *Wolbachia* infecting *K. gibbosae* (*w*Kgib), which was sequenced using third-generation *PacBio Sequel*, we used BLASTN against the data set of the genomes of known *Wolbachia* to find homologous reads of *Wolbachia* (identity ≥ 55%, coverage percent ≥ 55%, *e*-value ≤ 1e−5). The set of homologous reads was assembled using *Canu-1.7.1* (Koren et al., 2017), and finally *Purge Haplotigs* (Roach et al., 2018) was used for filtering the redundancy with low coverage in the assembly to get more concise and consecutive genome sequences.

The genomic integrality test for all three genomes was conducted using the Benchmarking Universal Single-Copy Orthologs (BUSCO v4.0.6) (Simao et al., 2015) with a proteobacteria database, and the integrality of the four genomes of *Wolbachia* known to be intact (*w*Ha, *w*Mel, *w*Au, and *w*CauA) was also tested for contrast. The genomes were then submitted to GenBank which would be automatically annotated by the *NCBI Prokaryotic Genome Annotation Pipeline (PGAP)* (Tatusova et al., 2016), with the accession numbers of CP054557 for *w*Wpum, CP054598 for *w*Csol and JABXYD000000000 for *w*Kgib.

### The Other *Wolbachia* Genomes Used in This Study

In addition to the above three *Wolbachia* in fig wasps, we also selected 49 known genomes of different strains of *Wolbachia* from GenBank for analysis. These strains were distributed in the supergroups A–F (Supplementary Table S1).

### Protein Prediction and Reannotation of the *Wolbachia* Genomes

To ensure the consistency of our data, we repredicted the encoding proteins from all the 52 strains of *Wolbachia*. Each genome was predicted using *Prodigal 2.6.3* (Hyatt et al., 2010). Then we reannotated all proteins of the *Wolbachia* strains in supergroup A with the following databases: NR database, Swiss-Prot, and InterProScan.

### Construction of a Phylogenetic Tree Based on Single-Copy Orthologous Proteins

*OrthoMCL* (Li et al., 2003) (with default parameters) was used to search for orthologous proteins among the 52 strains of *Wolbachia*. The set of single-copy orthologous proteins was obtained by manually excluding the multicopy orthologous proteins.

All single-copy orthologous proteins in each strain were concatenated in a specific order into a super-protein sequence and aligned using *MAFFT v7.313* (Katoh and Standley, 2013). Then we used *Gblocks 0.91b* (Talavera and Castresana, 2007) to select conserved blocks in the alignments. MrBayes (Altekar et al., 2004) was used to construct the phylogenetic tree of 52 super-proteins with the optimal amino acid substitution model predicted using *ProtTest 3.4.2* (Darriba et al., 2011).

### Search for Prophage WO in *Wolbachia* and Illustration of WO Structures

The location and number of prophage WO in each *Wolbachia* in supergroup A were ascertained through the combination of the annotation of genomes of *Wolbachia* (as obtained in this study) and the prediction from the platform *PHASTER* (Arndt et al., 2016). We uploaded the genomes of *Wolbachia* to PHASTER and compared them with the phage databases to obtain obscure positions of prophage WO. According to the functional annotation, the protein in the predicted position was confirmed to be the relative protein of prophage WO. If not, the protein was discarded, and if so, we continued to check the other proteins in its flanking region until the sequence consisted of non-WO genes at both ends. Then, the sequence contained in all these consecutive WO genes is designated as one prophage WO. The genomic structures of all prophages WO were illustrated using Illustrator for Biological Sequences (IBS) (Liu et al., 2015).

### Construction of Phylogenetic Tree and Estimation of Divergence Time of WO

We extracted the conserved sequences including the head module and baseplate module (from phage *terminase* to tail protein I) from each WO prophage found in the *Wolbachia* strains of supergroup A. The sequences were aligned, and the conserved blocks were selected as described above, based on which the phylogenetic tree was constructed using MrBayes with the prediction of the nucleotide substitution model using ModelFinder. All software involved was integrated into the PhyloSuite v1.2.1 software (Zhang et al., 2020).

To estimate the divergence time of prophages, we used BEAST v1.10.4 (Suchard et al., 2018) to construct the time tree, and the chain length of MCMC was set as 100,000,000. We simulated six trees using different models (Bayesian Skyline, Constant Size and Exponential Growth model in Uncorrelated relaxed clock type and Strict clock type, respectively) and chose the optimal tree based on the result of the PS/SS MLE test (Supplementary Table S2). Given that there was little fossil evidence to calibrate the divergence time of prophage WO, we referred to the method in the essay of William R. Corner, setting a relative time of the node between WOCsol and WOKgib as 1 to construct an arbitrary scaling time tree (Conner et al., 2017), to analyze the relative divergence order of prophages WO.

### Synteny Analyses of the Sequences

The synteny analyses were mainly performed using MCscanX (Wang et al., 2012) with the parameter of *e*-value ≤ 1e−10. In a few conditions, we employed *Mauve 2.4.0* (Darling et al., 2004) using its default parameters.

### RNA Extraction and Polymerase Chain Reaction Verification of Transcriptional Activity of Prophage WO Genes

For each species of fig wasp, we set eight samples (four of females and four of males). The individual numbers in each sample ranged from 30 to 50 according to the body size of the fig wasp (for *C. solmsi* and *W. pumilae*, there were 30 individuals per sample, whereas for *K. gibbosae*, there were 50 individuals per sample). We used TransZol Up Plus RNA Kit (TransGenBiotech, Beijing, China) to extract the RNA for each sample and finally dissolved them in 35 μL RNase-free water. The concentration and purity of RNA were tested according to the OD values using Thermo Scientific NanoDrop One. All RNA samples were used to performed PCR test with Wolbachia wsp81F/691R universal primers (Zhou et al., 1998) as the negative controls to confirm that they did not contain massive genomic DNA contamination. Then the cDNA was synthesized, and at the same time, the remaining genomic DNA contamination was removed using EasyScript^®^ II One-Step gDNA Removal and cDNA Synthesis SuperMix (TransGenBiotech, Beijing, China). The reaction mixture was incubated at 42°C for 30 min. We designed primers for PCR verification on the conserved functional genes of the prophages WO. To reduce the probability of false positives, we did not choose unspecific genes, such as *Ankyrin* repeats and *transposase*, which were probably also present in the insect and bacterial hosts. We verified 23 genes in WOCsol and WOKgib, and 15 genes in WOWpum (primers are listed in Supplementary Table S3). The expression of *wsp* gene in each cDNA sample was tested by PCR as the positive control to demonstrate the cDNA in all samples was well synthesized. The PCR products with bands of target sizes were sequenced to confirm the correct amplification of the target genes.

## Results

### Genome Assemblies and Supergroup Typing of *Wolbachia* Strains Infecting the Three Fig Wasp Species

We obtained the genome assemblies of three *Wolbachia* strains that infected the three fig wasp species. The genome of *Wolbachia* strain *w*Wpum was an intact sequence that could be circularized with the size of 1.28 Mb. The genome of *w*Csol consisted of a single scaffold with the size of 1.21 Mb, but it could not be circularized. The genome of *w*Kgib contained three scaffolds, with the total size of 1.45 Mb and N50 length of the assembly 948,431 bp. The integrality of each *Wolbachia* genome tested using BUSCO was *w*Wpum = 84.5%, *w*Csol = 83.6%, and *w*Kgib = 84.9%. Compared with the integrality of the other four intact *Wolbachia* strains (*w*Ha = 84%, *w*Mel = 84.5%, *w*Au = 84.9%, and *w*CauA = 84.5%), the genomes of *Wolbachia* infecting fig wasps did not present significant differences (*p* value of *w*Wpum = 1.000, *w*Csol = 0.147, and *w*Kgib = 0.264), indicating that our assemblies of genomes were sufficiently complete.

The assembling statistics of each *Wolbachia* infecting the fig wasps were annotated using PGAP and was compared with 49 other *Wolbachia* strains (Supplementary Table S4). The results showed few significant differences, whether compared with all strains or with the 24 strains in supergroup A, except in the case of *w*Kgib, where the numbers of rRNA (*n* = 6, *p* = 0.026 < 0.05 to all strains) and ncRNA (*n* = 5, *p* = 0.002 < 0.01 to all strains, and *p* = 0.001 < 0.01 to supergroup A) increased significantly (Table 1).

**TABLE 1:** Mann-Whitney U tests for assembling statistics of each Wolbachia in fig wasp against other strains.

	*w*Wpum vs. all strains	*w*Csol vs. all strains	*w*Kgib vs. all strains	*w*Wpum vs. supergroup A	*w*Csol vs. supergroup A	*w*Kgib vs. supergroup A
Size	0.917	0.488	0.282	0.889	0.366	0.126
GC%	0.327	0.972	0.727	0.711	0.165	0.379
Protein	0.51	0.425	0.177	0.488	0.267	0.127
rRNA	0.828	0.828	0.026*	0.853	0.853	0.115
tRNA	0.108	0.846	0.072	0.14	1	0.104
ncRNA	0.758	0.758	0.002**	0.827	0.827	0.001**
Gene	0.652	0.386	0.225	0.782	0.267	0.127
Pseudogene	0.912	0.883	0.658	0.693	0.693	0.478

The numbers in the cells represent the p-values. *p < 0.05, **p < 0.01.

**FIGURE 1:**
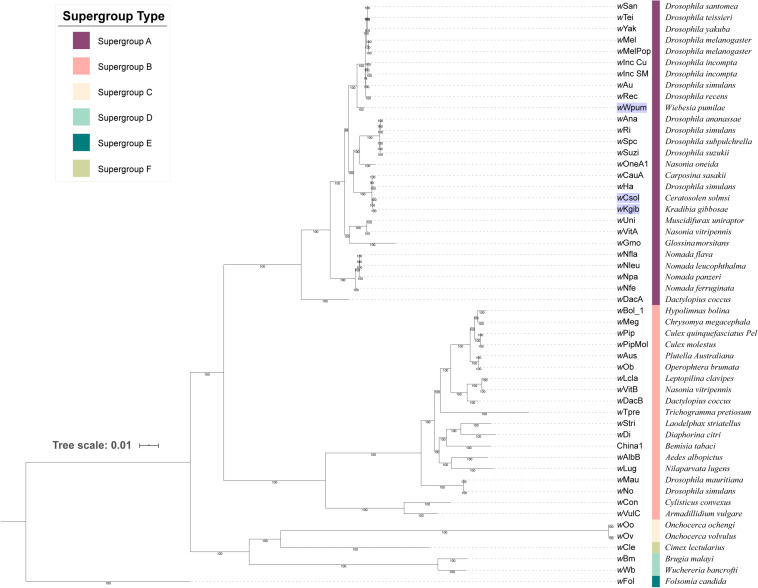
Phylogeny of 52 strains of *Wolbachia*. The tree was constructed based on 253 groups of single-copy orthologous proteins using MrBayes and the amino acid substitution model CpRev + I + G + F, and *w*Fol was set as the outgroup. The three *Wolbachia* strains infecting fig wasps were marked with a violet background. Each supergroup was labeled using different color stripes, and after the stripes, the eukaryotic host that each *Wolbachia* strain belongs to was listed.

Based on 253 groups of single-copy orthologous proteins, we constructed a Bayesian phylogenetic tree with 52 strains of *Wolbachia* (Figure 1). It revealed that all the three *Wolbachia* strains in fig wasps were classified into supergroup A, with the strains of *w*Csol and *w*Kgib clustered together to form a single branch, whereas the strain of *w*Wpum showing a closer relationship with several *Wolbachia* strains that infect fruit flies on another branch.

### Structural Diversity of WO Prophages in the *Wolbachia* Strains in Supergroup A

**FIGURE 2:**
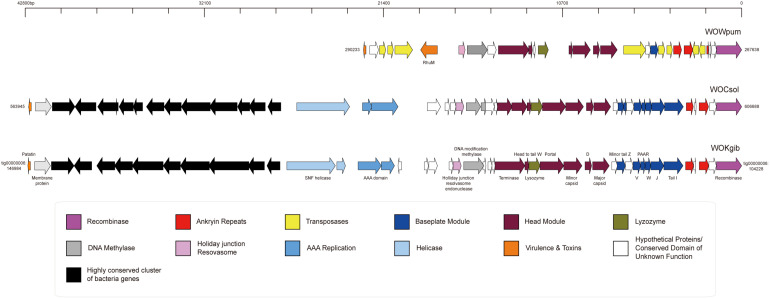
Genomic structures of the three cryptic WO prophages in *Wolbachia* found in fig wasps. The structures of the core regions of the prophages were illustrated by IBS, and the functional annotation of genes was labeled. In WOWpum, the highly conserved cluster of bacterial genes was absent, and quite a few genes of the head module, baseplate module, AAA replication, and helicase were missing or degraded.

Based on the three well-assembled genomic sequences of *Wolbachia* strains in the fig wasps, we searched for and annotated the sequences of prophages WO and noticed that each *Wolbachia* strain possessed merely one prophage WO, named as WOCsol, WOKgib, and WOWpum, respectively, corresponding to the *Wolbachia* strains of *w*Csol, *w*Kgib, and *w*Wpum (Figure 2). The length of WOCsol was 42,744 bp, the length of WOKgib was 42,757 bp, and WOWpum was 22,596 bp. Interestingly, no gene member of the tail module was found in any of the three prophages. The defect in the genomic structure indicated that these WO were cryptic phages with no capacity to form virions. To the best of our knowledge, it is the first time that cryptic prophages WO existing independently without any intact prophage WO in the *Wolbachia* strains among a group of insect hosts is being reported. WOCsol and WOKgib were very similar in terms of structure and sequence length, and the similarity of nucleotide sequences was 99.82%. However, compared with WOCsol or WOkgib, the sequence of WOWpum was almost half the length, which was owing to the lack of insertion of a highly conserved cluster of bacteria genes and the absence of genes of other modules, including that of the head, baseplate, helicase, and AAA replication. These results suggested a relatively distant relationship between WOWpum and the other two WOs infecting fig wasps, which seemed to be consistent with the phylogeny of their bacterial host, *Wolbachia*.

**FIGURE 3:**
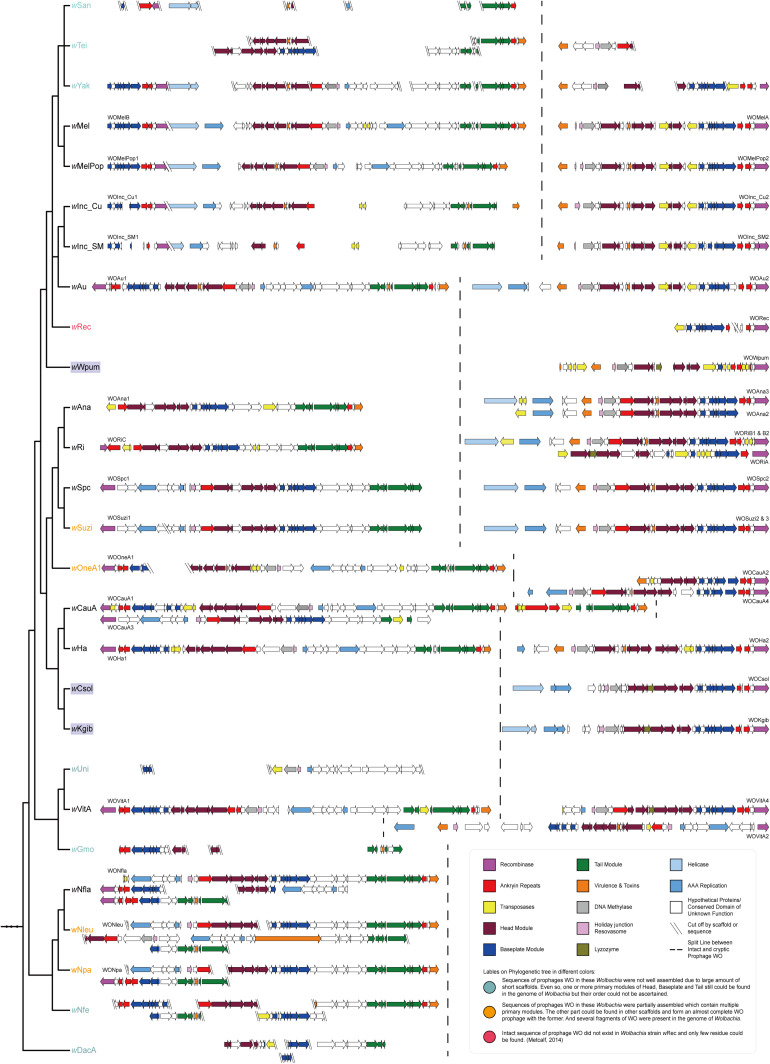
Mapping of the genomic structures of WO in 27 *Wolbachia* strains in supergroup A to the phylogenetic tree of their bacterial hosts. In each of the *Wolbachia* strains, there were different numbers of cryptic WO prophages with at least one intact prophage WO, but exceptionally, each of the three strains infecting fig wasps possessed only one cryptic prophage WO without the existence of any intact prophage WO. The dashed line was used for separating intact WO (on the left) and cryptic WO (on the right) sequences. Phylogenetic tree of *Wolbachia* was the same as the section of supergroup A in Figure 1, ignoring branch length.

To determine whether it is a special phenomenon that *Wolbachia* that infect fig wasps contain only cryptic prophages without the existence of any intact WO, as well as to understand the tendency of structural change and the evolutionary relationship of the WO prophages, we searched for and annotated the prophages WO that infect the other 24 *Wolbachia* strains in supergroup A. We then mapped the structures of the WO onto the phylogeny of *Wolbachia* (Figure 3). The results showed that except the three *Wolbachia* that infected the three fig wasps studied and the previously reported *w*Rec, each *Wolbachia* in supergroup A possessed at least one intact WO. Even though some prophages were not well assembled because of the severe fragile assemblies of their bacterial host, the sequence fragments of the head, baseplate, and tail modules (*w*San, *w*Tei, *w*Yak, *w*Gmo, *w*Nfe, *w*DacA) or the homologous fragments of other intact WO (*w*Uni) could still be found, implying the presence of intact prophages in these *Wolbachia* strains. Furthermore, simply in terms of structure, within a *Wolbachia* strain, the intact WO is usually quite different from the cryptic WO, while the intact prophages WO or cryptic prophages WO among *Wolbachia* strains with close relationships were much more similar. Therefore, we reasonably speculated that the cryptic WO of each *Wolbachia* strain was not derived directly from the intact WO of the same *Wolbachia* strain.

### Phylogeny and Estimated Divergence Time of the WO Prophages

To verify the above speculation, we constructed an arbitrary scaling divergence time tree and phylogenetic tree for all prophages referred to in the previous section (the prophages without consecutive sequences were excluded), based on their conserved nucleotide sequences of the head and baseplate modules.

**FIGURE 4:**
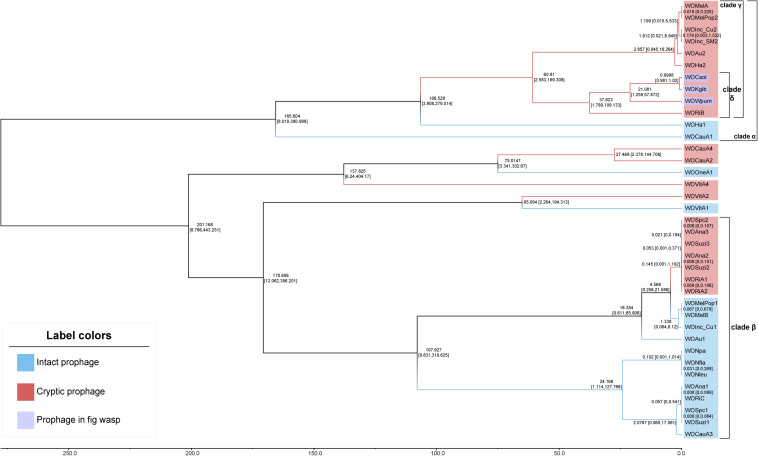
Chronograms for WO prophages. The chronogram depicted a relative divergence time of each WO prophage to the node of WOCsol–WOKgib, where we set an arbitrary scaling of relative age at 1. The estimated divergence time of each node is shown with 95% confidence intervals. Branches and labels of this unrooted tree are shown in two diverse colors to distinguish between the intact and cryptic prophages. In both of clade α and clade β, intact WOs usually appeared earlier than cryptic WOs. Clade γ was a typical clade in which all prophages were cryptic. All four WOs in clade δ were found to have genes with transcriptional activities.

The divergence time tree (Figure 4) showed that intact WO was unsurprisingly more ancient than cryptic WO. For most of the phages, such as those in clade α and clade β, all cryptic WOs were newly produced after intact WOs. Furthermore, the estimated divergence time of the WO prophages of *Wolbachia* strains infecting *Drosophila* (WOSpc, WOAna, WOSuzi, and WORi) and *Nomada* (WONpa, WONfla, and WONleu) in clade β was very late, even later than the time set as 1 for the node between WOCsol and WOKgib. In the *Wolbachia* strains of *Drosophila*, the divergence time of both cryptic and intact WO was extremely close, and even the estimated divergence time was exactly the same (in 0.008 or 0.009 relative time), such as in the WO of *w*Spc, *w*Ana, *w*Suzi, and *w*Ri, revealing a clear pattern of coevolution and codivergence. Surprisingly, WOWpum was clustered with WOCsol/WOKgib, showing a very close phylogenetic relationship, rather than the greater distance between their bacterial hosts. However, the divergence time of WOWpum was indeed earlier than the other two WOs, indicating that more selection was carried out in its case, which was consistent with its more fragmented genome structure as mentioned above.

**FIGURE 5:**
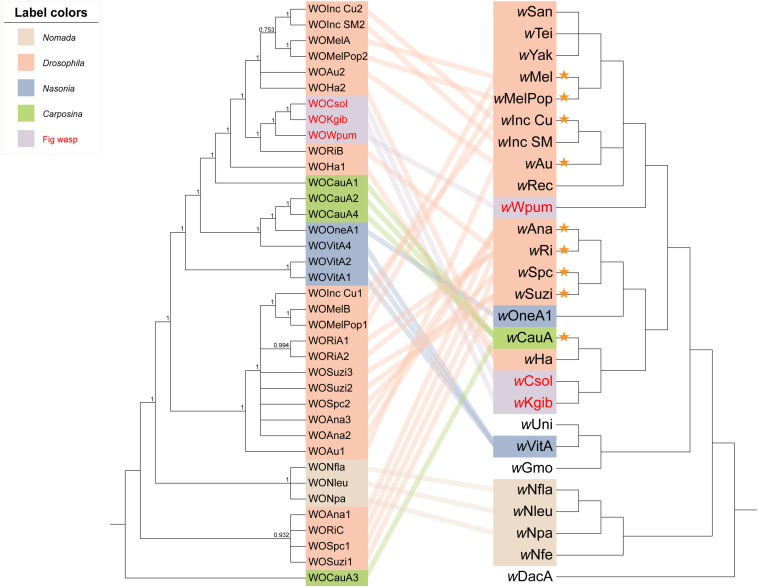
Phylogenetic relationships of the WO prophages, *Wolbachia* strains, and insect hosts. The tree of WO on the left was constructed using MrBayes in the nucleotide substitution model GTR + F + I + G4, and WOCauA3 is set as the outgroup. The tree on the right is the same as the section depicting supergroup A of *Wolbachia* as shown in Figure 1, in which the branch length is ignored. We connected each prophage to their bacterial host with lines, the colors of which labeled the insect host group. The WO/*Wolbachia* in the same color belonged to one specific genus or a biological group of insects. Orange asterisks represent the *Wolbachia* strains with the intact and cryptic WO relatively far apart.

When we integrated the phylogeny of the WO prophages, *Wolbachia*, and their insect hosts together to form an intuitive relationship among them (Figure 5), the results showed that (a) the phenomenon that prophages WO among distant *Wolbachia* strains presented close relationships not only appeared in the group of fig wasps, but also in the insect hosts of the Hymenoptera genus of *Nasonia*. For example, the *Wolbachia* strain *w*OneA1 infecting *Nasonia oneida* was apparently evolutionarily distant from the strain infecting *Nasonia vitripennis*, *w*VitA, but their prophages WOVitA4 and WOOneA1 were relatively close and were even clustered into one clade. (b) However, in the genus of *Drosophila*, the topological structure of the phylogeny of *Wolbachia* (except for *w*Ha and *w*Au) was pretty similar to that of their prophages, such as the clade of *w*Mel: *w*Au and the clade of *w*Ana: *w*Suzi. This relationship was also seen in the Hymenoptera genus of *Nomada*. (c) Interestingly, consistent with our speculation that in the same *Wolbachia* strain, the cryptic WO is not similar to the intact WO, except for in the case of WOVitA and WOHa, a few cryptic WO (in 9/20 of *Wolbachia* strains, which are labeled using orange asterisks) were evolutionarily dissimilar to the intact WO in the same strain. Moreover, if we counted only the *Wolbachia* strains possessing both intact and cryptic WO, the percentage would rise to 82% (9/11), indicating a substantial probability that cryptic prophages WO were not derived directly from the intact ones in the same bacterial host.

### High Proportion of Genes of the WO Prophages Were Actively Transcribed in the Fig Wasps

**FIGURE 6:**
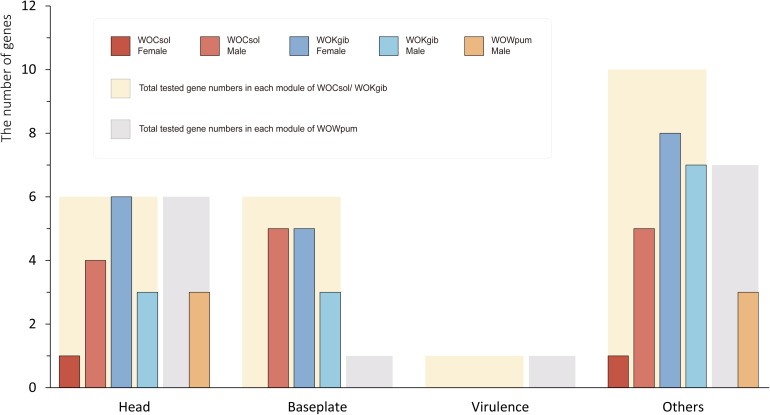
Sexual-specific and module-specific transcription activities of genes in the three WO prophages. The numbers of genes of the prophages verified by RT-PCR were counted. The thick bars in the background represent the total numbers of genes for which we designed primers for amplification, and the narrow bars represent the actual numbers of genes possessing transcriptional activities in each WO sample. Results of six WO samples were split into four groups according to the different functional modules. WOCsol and WOWpum showed a higher transcription activity in males than that in females, and in WOKgib, the pattern was observed to be the opposite. Meanwhile, the genes of head modules of the three WOs were transcribed to a greater extent than those of the baseplate modules, which showed functional module preference on conserved modules.

We verified the transcriptional activity of each characteristic functional gene in the core region of the WO prophages of the respective fig wasps using reverse transcription PCR (RT-PCR). Twenty-three genes were verified in WOCsol and WOKgib, respectively, and 15 genes were verified in WOWpum. In WOCsol, only two genes showed transcription in females, whereas 14 genes were active in males, with the percentage of actively transcribed genes at 61% (14/23). In WOKgib, 19 genes were transcribed in females, and 13 were transcribed in males, with the percentage of actively transcribed genes at 83% (19/23). In WOWpum, the mRNA of these genes was not detected in females, whereas in males, six genes showed transcriptional activity, with the percentage of active genes at 40% (6/15) (Table 2). Thus, these actively transcribed WO genes showed a certain gender preference in the fig wasp hosts (Figure 6), with WOCsol and WOWpum having significantly more actively transcribed genes in wasp males than in females, while WOKgib having more actively transcribed genes in the female host. Furthermore, the functional module preference could also be discovered, although the genes of the baseplate and head modules were both conserved, genes of the head module showed more activity than that of the baseplate module. For the genes of the baseplate module, only the genes of WOCsol were transcribed actively in male hosts, and the genes of WOKgib were transcribed in both genders of the host, whereas in other samples, they showed no activity (in WOCsol-F, WOWpum-F, and WOWpum-M). In contrast, genes of the head module showed high transcriptional ratios in four samples, except for the samples of WOCsol-F (one active gene) and WOWpum-F (no active gene).

**TABLE 2:** RNA transcription of the three cryptic WO prophage in fig wasps.

Module	Gene	C-F	C-M	K-F	K-M	W-F	W-M
Others	Site-specific recombinase	−	+	+	+	−	+
Others	Putative phage−related protein	−	+	+	+	−	+
Baseplate	Tail I	−	+	+	−		
Baseplate	Baseplate assembly protein J	−	−	+	−		
Baseplate	Prophage lambda W1, baseplate assembly protein W	−	+	−	−		
Baseplate	PAAR motif	−	+	+	+		
Baseplate	Baseplate assembly protein GpV	−	+	+	+		
Others	Hypothetical protein So0009	−	+	+	−		
Baseplate	Prophage minor tail protein Z (GPZ)	−	+	+	+	−	−
Others	Hypothetical protein So0011	+	+	+	+	−	+
Head	Putative major capsid protein	−	+	+	−	−	+
Head	Bacteriophage lambda head decoration protein D	+	+	+	+	−	−
Head	Putative minor capsid protein C	−	−	+	−	−	+
Head	Phage portal protein	−	+	+	+	−	−
Others	Lyzozyme M1	−	−	+	+	−	+
Head	Head-to-tail joining protein W	−	+	+	+	−	−
Head	Phage terminase large subunit family protein	−	−	+	−	−	−
Others	Hypothetical protein So0019	−	+	+	+	−	−
Others	DNA modification methylase	−	−	−	−	−	−
Others	Putative Holliday junction resolvasome endonuclease	−	−	+	+	−	−
Others	DEAD/DEAH box helicase	−	−	−	−		
Others	Putative membrane protein	−	−	+	+		
Virulence	Patatin-like phospholipase	−	−	−	−		
Virulence	Virulence RhuM family protein					−	−

**The abbreviation of each sample’s name: C, WOCsol; K, WOkgib; W, WOWpum; M, male; F, female.The plus sign (+) represents the gene expression is positive in at least two of four samples. The minus sign (−) represents no gene expression is characterized in all four samples.**

## Discussion

In this study, based on the three well-assembled genome sequences of the *Wolbachia* strains of *w*Wpum, *w*Kgib, and *w*Csol that infect three fig wasp species, we ascertained the numbers and structural features of the temperate WO prophages. Each of these three strains contained only one typical cryptic prophage WO, which contained the head and baseplate modules, but no gene of the tail module. However, we found no intact prophages capable of forming active virions in these *Wolbachia* strains, which was consistent with previous results that the *Wolbachia* strain infecting fig wasp *C. solmsi* merely had one cryptic prophage ascertained using the method of q-PCR (Wang et al., 2013, 2014). Furthermore, we explored the distribution of the WO prophages of the previously reported 24 *Wolbachia* strains in supergroup A and found that the phenomenon of only one cryptic prophage existing without an intact prophage WO was unique to the *Wolbachia* strains of fig wasps. This result was contrary to the widely accepted understanding that *Wolbachia* with cryptic prophages usually possesses at least one intact WO prophage (Kent et al., 2011). It is worth mentioning that although the *Wolbachia* strain of *w*Rec possessed only fragmented sequences of WO (Metcalf et al., 2014), the assembly of the *w*Rec genome was not consecutive. Therefore, the detection and analysis of WO may be biased, and many of the WO sequences might have been discarded during the assembly process. In contrast, in the present study, the assemblies of the *Wolbachia* genomes in fig wasps are much more complete, which would be more convincing to indicate the absence of an intact WO and the existence of a single cryptic WO in the bacterial hosts. To the best of our knowledge, this is the first report describing the phenomenon of a cryptic WO prophage existing solely in the absence of an intact prophage in the *Wolbachia* strains of a specific group of insect hosts.

We reannotated and illustrated the genomic structures of the core regions of all the WO prophages of *Wolbachia* in supergroup A involved in this study. Simultaneously, we constructed a phylogenetic tree and an arbitrary scaling divergence time tree based on the relatively conserved sequences ranging from the head to the baseplate modules commonly found in WO, and these phylogenetic trees were then mapped to the phylogenetic tree of their bacterial hosts. Both the structural features and phylogenetic results indicated that most of the cryptic prophages WO were not derived from the intact ones in the same *Wolbachia* strain; instead, their relationship was relatively distant. In addition, as the phages of *Wolbachia*, WOs showed a close relationship when the bacterial hosts were relatively distant, and interestingly, the insect hosts of the prophages with this feature were usually from the same genus or the same group of organisms. These results indicate that WO could be actively transmitted horizontally among different *Wolbachia* strains. WO has been reported to be able to transmit horizontally between different *Wolbachia* strains (even belonging to different supergroups) coinfected in the same host cell (Bordenstein and Wernegreen, 2004; Kent et al., 2011). Our results were expanded on this basis, and we believed that WO could be transmitted horizontally among a certain genus or a group of organisms and even spread across species over long distances driven by certain unknown factors (such as the spread of WOs between fruit fly and fig wasps in clade δ in Figure 4). Of course, the evidence of the codivergence of WO in the *Drosophila* genus suggested that WO could also be transmitted vertically in the *Wolbachia* host. Combined with the evidence that the estimated divergence time between *w*Spc and *w*Suzi was between 1,000 and 10,000 years ago (Conner et al., 2017), it can be inferred that the vertical transmission of WO might be a recent event compared with the event of horizontal transmission. In summary, we speculate that the rapid horizontal transmission of WO among a wide range of eukaryotic hosts and vertical transmission along with newly divergent *Wolbachia* strains could explain the wide distribution of WO phages in *Wolbachia*, and the horizontal (Conner et al., 2017) or vertical transmission (Werren et al., 2008) of *Wolbachia* could further promote the spread of WO among eukaryotic hosts.

Besides, we could draw more inferences or speculations based on the above results to elucidate the selection undergone by the WO phages and their evolutionary path after insertion into their *Wolbachia* hosts. (a) The genes of the four prophages WO in clade δ (in Figure 4), including the three WOs in fig wasps and WORiB, were all detected to have different levels of transcriptional activities, and the more recently WO diverged, the more genes with transcriptional activities it preserved. These discoveries verified that the prophages could be inactivated gradually by their *Wolbachia* hosts. Therefore, we reasonably inferred that the ancestor of clade γ was possibly an intact prophage WO with horizontal transmission capability in its virion form when it diverged from its Most Recent Common Ancestor, along with WOHa1 at the relative time of 106.528. After horizontal transmission across multiple *Wolbachia* strains, all the WOs derived from the ancestor WO phages became cryptic, explaining why several cryptic prophages WO were in the clade γ. (b) Given that *Wolbachia* was able to affect the WO phages negatively by causing the degradation and inactivation of their genes, WOs may also resist the selective pressures of their *Wolbachia* hosts. We noticed that the genes of both WOCsol and WOWpum presented higher transcriptional activities in male hosts than in females, which showed a certain gender preference. As *Wolbachia* is an endosymbiont with maternal transmission, it meant that the bacteria infecting males cannot be inherited by descendants. Considering the “non-compliance” of WOs to their *Wolbachia* hosts and the congruence between the phylogeny of WOs and the groups of insect hosts, we suggested two possible explanations for the higher transcription of WO phage genes in male hosts than in females. First, in the male insect host, WOs may increase the gene transcription to activate its horizontal transmission as quickly as possible, in order to escape the dead end of vertical transmission in the male insect host. Second, in the tripartite symbiont of insect-*Wolbachia*-WO, male insect hosts might tend to align with the prophages to antagonize the *Wolbachia* strain, so prophages WO could show higher activity in male insects where the *Wolbachia* strain is relatively “weak.” In the coevolution between the *Wolbachia* hosts and prophages, we could also observe the antagonism between them. In the analysis of the cryptic WOs, genes of the head module showed higher transcriptional activities than those of the baseplate module, which revealed that the genes of head module might be more crucial for WO. Combined with the absence of the tail modules in the cryptic prophages, we could speculate that under the selective pressure exerted by *Wolbachia*, WOs would be degraded in the order of the functional modules of the tail, baseplate, and head, which may be to maximize the retention time of its activity.

## Data Availability Statement

The datasets presented in this study can be found in online repositories. The names of the repository/repositories and accession number(s) can be found in the article/Supplementary Material.

## Author Contributions

Y-hM, J-hX, and D-wH designed the study. Y-hM performed the experiments. Y-hM and J-hX analyzed the data and wrote the manuscript. All authors have read and approved the submitted manuscript.

## Conflict of Interest

The authors declare that the research was conducted in the absence of any commercial or financial relationships that could be construed as a potential conflict of interest.
