# Supplementary material for: Distribution and Evolution of the Bacteriophage WO and Its Antagonism With Wolbachia
Source: Front Microbiol. 2020 Nov 13;11:595629. doi: 10.3389/fmicb.2020.595629 (PMC7691483; doi:10.3389/fmicb.2020.595629)
Supplement: Supplementary file 1 [file Table_1.DOCX]

**Supplementary Table 1.** Genomes used for analysis in the study

| Supergroup | Strain | Accession | Host |
| --- | --- | --- | --- |
| A | *w*San | NZ_VCEH00000000.1 | *Drosophila santomea* |
| A | *w*Tei | NZ_VCEG00000000.1 | *Drosophila teissieri* |
| A | *w*Yak | NZ_VCEF00000000.1 | *Drosophila yakuba* |
| A | *w*Mel | NC_002978.6 | *Drosophila melanogaster* |
| A | *w*MelPop | NZ_AQQE00000000.1 | *Drosophila melanogaster* |
| A | *w*Inc_Cu | CP011148.1 | *Drosophila incompta* |
| A | *w*Inc_SM | CP011149.1 | *Drosophila incompta* |
| A | *w*Au | NZ_LK055284.1 | *Drosophila simulans* |
| A | *w*Rec | NZ_JQAM00000000.1 | *Drosophila recens* |
| A | *w*Ana | NZ_CP042904.1 | *Drosophila ananassae* |
| A | *w*Ri | CP001391.1 | *Drosophila simulans* |
| A | *w*Spc | NZ_NTHL00000000.1 | *Drosophila subpulchrella* |
| A | *w*Suzi | NZ_CAOU00000000.2 | *Drosophila suzukii* |
| A | *w*OneA1 | NZ_QESS00000000.1 | *Nasonia oneida* |
| A | *w*CauA | CP041215.1 | *Carposina sasakii* |
| A | *w*Ha | NC_021089.1 | *Drosophila simulans* |
| A | *w*Uni | NZ_MUJL01000001.1 | *Muscidifurax uniraptor* |
| A | *w*VitA | NZ_MUJM01000001.1 | *Nasonia vitripennis* |
| A | *w*Gmo | NZ_AWUH00000000.1 | *Glossina morsitans* |
| A | *w*Nfla | NZ_LYUW00000000.1 | *Nomada flava* |
| A | *w*Nleu | NZ_LYUV00000000.1 | *Nomada leucophthalma* |
| A | *w*Npa | NZ_LYUX00000000.1 | *Nomada panzeri* |
| A | *w*Nfe | NZ_LYUY00000000.1 | *Nomada ferruginata* |
| A | *w*DacA | NZ_LSYX00000000.1 | *Dactylopius coccus* |
| B | *w*Bol1-b | NZ_CAOH00000000.1 | *Hypolimnas bolina* |
| B | *w*Meg | CP021120.1 | *Chrysomya megacephala* |
| B | *w*PipPel | NC_010981.1 | *Culex quinquefasciatus Pel* |
| B | *w*PipMol | NZ_CTEH00000000.1 | *Culex molestus* |
| B | *w*Aus | NZ_MRWX01000001 | *Plutella Australiana* |
| B | *w*Ob | NZ_JYPC00000000.1 | *Operophtera brumata* |
| B | *w*Lcla | NZ_QJHA00000000.1 | *Leptopilina clavipes* |
| B | *w*VitB | NZ_AERW00000000.1 | *Nasonia vitripennis* |
| B | *w*DacB | NZ_LSYY00000000.1 | *Dactylopius coccus* |
| B | *w*Tpre | CM003641.1 | *Trichogramma pretiosum* |
| B | *w*Stri | NZ_MUIX00000000.1 | *Laodelphax striatellus* |
| B | *w*Di | NZ_AMZJ01000001.1 | *Diaphorina citri* |
| B | China 1 | CP016430.1 | *Bemisia tabaci* |
| B | *w*AlbB | CP031221.1 | *Aedes albopictus* |
| B | *w*Lug | NZ_MUIY00000000.1 | *Nilaparvata lugens* |
| B | *w*Mau | CP034335.1 | *Drosophila mauritiana* |
| B | *w*No | NC_021084.1 | *Drosophila simulans* |
| B | *w*Con | NZ_QPIP00000000.1 | *Cylisticus convexus* |
| B | *w*VulC | NZ_ALWU01000001.1 | *Armadillidium vulgare* |
| C | *w*Oo | NC_018267.1 | *Onchocerca ochengi* |
| C | *w*Ov | NZ_HG810405.1 | *Onchocerca volvulus* |
| D | *w*Bm | NZ_CP034333.1 | *Brugia malayi* |
| D | *w*Wb | NZ_NJBR00000000.2 | *Wuchereria bancrofti* |
| E | *w*Fol | CP015510.2 | *Folsomia candida* |
| F | *w*Cle | NZ_AP013028.1 | *Cimex lectularius* |

**Supplementary Table 2.** Result of PS/SS MLE tests for divergence time trees in six different models

| Clock type | Model | PS | SS |
| --- | --- | --- | --- |
| Uncorrelated relaxed clock | Bayesian Skyline | -78446.9 | -78447.092 |
|  | Constant Size | -79018.909 | -79016.738 |
|  | Exponential Growth | -78469.624 | -78465.427 |
| Strict clock | Bayesian Skyline | -79722.677 | -79722.377 |
|  | Constant Size | -79765.429 | -79764.374 |
|  | Exponential Growth | -79771.062 | -79769.825 |

**Supplementary Table 3.** Conserved genes of three prophages WO infecting fig wasps and their primers for RT-PCR

| No. | gene | direction | Primer for WOCsol/ WOKgib | Primer for WOWpum |
| --- | --- | --- | --- | --- |
| 1 | site-specific recombinase | F | AAGGGTGGGTAGCATTAGC | AAGGGTGGGTAGCATTAGC |
|  |  | R | ACCACGCAGTCTACCTCTCC | ACCACGCAGTCTACCTCTCC |
| 2 | putative phage related protein | F | AACTGCCAGTAGAGGGAACAG | GCGTATAGGTTGCAGGAGAA |
|  |  | R | AGGACCGTTGTAGCTCATTCC | TACCGCATGCGTTTCCTCAC |
| 3 | tail I | F | GCCAGCAAGATCGAGGCTAA |  |
|  |  | R | GCCGGTAGTAAGCTCTGTGG |  |
| 4 | baseplate assembly protein J | F | GAAGTAGCAGCATGGCGAGA |  |
|  |  | R | CCCTGGTGACTTGCTTTCCT |  |
| 5 | prophage LambdaW1, baseplate assembly protein W | F | AGGGATGAGCAAAGAAACAGG |  |
|  |  | R | ACGTGATCCTATCGGTGTGG |  |
| 6 | PAAR motif | F | TGCAGTGGAATACCAGCACAT |  |
|  |  | R | CGCTGTTTGATCCTTGCGTT |  |
| 7 | baseplate assembly protein GpV | F | TGAAAAAGCAAGAGTGCGGG |  |
|  |  | R | ATTCGGCGCAAACCAACTTC |  |
| 8 | hypothetical protein So0009 | F | AACAGAGGAATTGGCGCTGA |  |
|  |  | R | TGCACCCATTCAACCATCCA |  |
| 9 | Prophage minor tail protein Z (GPZ) | F | AGGCGTCAGGATGCGTATAAA | GTGGAAAAAGCAACGGTGAGG |
|  |  | R | TGCTTCTGGCTCCAATGCTAA | CCTGCCCTTGCTCCTATCTTT |
| 10 | hypothetical protein So0011 | F | TTAGGGCAGCAAGCGTTGTA | GGACAGCAAGCTTTGTATGAG |
|  |  | R | GCATCCTGACGCCTGAATCT | ACACCCCTGACGCTTGAA |
| 11 | putative major capsid protein | F | AGCGTTGGCTAACGTGGTAA | AGCAGCGCTAAGGAACGA |
|  |  | R | TTCCTTAGCGCTGCTCCTTC | AACAGTGGCCCGTGTCTTT |
| 12 | Bacteriophage lambda head decoration protein D | F | TAAACGCAACGGAAACCACC | GGGGCAAAACCTAAAGCTTGG |
|  |  | R | CTGCAGCTTTTTGCTCCTCAG | CCTATCGCTGTTTGCGTTCC |
| 13 | putative minor capsid protein C | F | GCCCCGGAGGAGAAGTAAAC | TCCGCATGAGCCGATAACA |
|  |  | R | ATACTTCCTTGCGGCACCTC | CTTCCTTGCGGCACCTCTC |
| 14 | phage portal protein | F | CGAGGAGTACCGTGGCTTTC | GGGGCAAAGAGAGGCGTA |
|  |  | R | GCTACCCCTTGCTCATTGCT | GCACCTCCGGCGAAACT |
| 15 | lyzozyme M1 | F | GGTACACCAGGGATGCTTGTA | TGGCATAGGAGCAAACGGA |
|  |  | R | GTACTTCACCCCTTGCGTTG | TCAGTGGCAAACGACCTGT |
| 16 | Head-to-tail joining protein W | F | AAAGAAGCTACAGAGCGGGG | AGCTACAGAGCGGGGGAA |
|  |  | R | GGCTTCACACCTGCGACTTT | ATCCTGCGCTTCGGCTTT |
| 17 | phage terminase large subunit family protein | F | ATAGGCGGAACAGAAGCTGG | ACCAAACACCGGGACGAA |
|  |  | R | GCAAACGTAGTGGGCTGTTG | CCGGCATAGAGCGCAGAC |
| 18 | hypothetical protein So0019 | F | GCAAGGGAACAAGGGTTTGC | AGGAGGAGGGGCGAAAGT |
|  |  | R | AGTTTTCCCCGCTCCATCTC | TCCCCGCTCCATCTCGTT |
| 19 | DNA modification methylase | F | AGCTAACTGGGCAAAGTGGG | GTGCGCCTCTATTCGGGA |
|  |  | R | GCAGTGAGTTGTACGTTGGC | TCGACCAACGACCACCTG |
| 20 | putative holliday junction resolvasome endonuclease | F | AGGTACTGACGCTGCACATT | TGCTCATTGCTACGGAGGC |
|  |  | R | ACACAAAGCATCAACGCCAA | ACTTGCATTGCCCTTACCG |
| 21 | DEAD/DEAH box helicase | F | GCAACTGTCCTGATTGGGCT |  |
|  |  | R | CAACGCCGGAATCCACCTAA |  |
| 22 | putative membrane protein | F | TTTTCCACTGTGCCTTTGCTG |  |
|  |  | R | GCAGTAGCATCTGCAAGAGTA |  |
| 23 | Patatin-like phospholipase | F | AAGTAGCATCAGCTGAGATGG |  |
|  |  | R | GCATTTGCCTCCTGCTGC |  |
| 24 | virulence RhuM family protein | F |  | TACGAGCATCCGAACGTAGAT |
|  |  | R |  | CAGCCAGGGCTTTTGCAACT |

**Supplementary Table 4.** Annotation statistics of 52 *Wolbachia* strains by PGAP

| Strain | Size (Mb) | GC% | Protein | rRNA | tRNA | ncRNA | Gene | Pseudogene |
| --- | --- | --- | --- | --- | --- | --- | --- | --- |
| wWpum | 1.28 | 35.2 | 1157 | 3 | 37 | 4 | 1332 | 131 |
| wCsol | 1.21 | 35.0 | 1003 | 3 | 34 | 4 | 1176 | 132 |
| wKgib | 1.45 | 35.1 | 1261 | 6 | 44 | 5 | 1475 | 159 |
| wSan | 1.18 | 35.1 | 1087 | 11 | 34 | 4 | 1197 | 61 |
| wTei | 1.30 | 35.3 | 1106 | 10 | 40 | 4 | 1267 | 107 |
| wYak | 1.29 | 35.1 | 1113 | 3 | 34 | 4 | 1260 | 106 |
| wMel | 1.27 | 35.2 | 1116 | 3 | 34 | 4 | 1286 | 129 |
| wMelPop | 1.24 | 35.2 | 1029 | 3 | 34 | 4 | 1173 | 103 |
| wInc_Cu | 1.27 | 35.8 | 917 | 3 | 33 | 4 | 1232 | 275 |
| wInc_SM | 1.27 | 36.4 | 557 | 2 | 31 | 1 | 1170 | 593 |
| wAu | 1.27 | 35.2 | 1099 | 3 | 34 | 4 | 1265 | 125 |
| wRec | 1.13 | 35.1 | 965 | 3 | 34 | 4 | 1133 | 127 |
| wAna | 1.40 | 35.2 | 1218 | 3 | 35 | 4 | 1367 | 107 |
| wRi | 1.45 | 35.2 | 1245 | 3 | 35 | 4 | 1409 | 122 |
| wSpc | 1.42 | 35.7 | 1239 | 42 | 54 | 4 | 1438 | 99 |
| wSuzi | 1.42 | 35.2 | 1248 | 3 | 35 | 4 | 1423 | 133 |
| wOneA1 | 1.29 | 32.8 | 1054 | 3 | 31 | 4 | 1201 | 109 |
| wCauA | 1.45 | 35.0 | 1260 | 3 | 34 | 4 | 1435 | 134 |
| wHa | 1.30 | 35.1 | 1123 | 3 | 34 | 4 | 1268 | 104 |
| wUni | 1.05 | 35.2 | 1174 | 2 | 31 | - | 1207 | - |
| wVitA | 1.21 | 34.9 | 1456 | 3 | 32 | - | 1491 | - |
| wGmo | 1.02 | 35.1 | 800 | 3 | 34 | - | 837 | - |
| wNfla | 1.33 | 35.2 | 1090 | 3 | 34 | 4 | 1354 | 223 |
| wNleu | 1.37 | 35.2 | 1123 | 3 | 34 | 4 | 1382 | 218 |
| wNpa | 1.34 | 35.2 | 1102 | 3 | 35 | 4 | 1372 | 228 |
| wNfe | 1.34 | 35.2 | 1078 | 3 | 34 | 4 | 1364 | 245 |
| wDacA | 1.17 | 35.1 | 1040 | 4 | 31 | 4 | 1202 | 123 |
| wBol1-b | 1.38 | 33.9 | 1152 | 3 | 34 | 4 | 1297 | 104 |
| wMeg | 1.38 | 34.0 | 1136 | 3 | 34 | 4 | 1302 | 125 |
| wPipPel | 1.48 | 34.2 | 1256 | 3 | 34 | 4 | 1425 | 128 |
| wPipMol | 1.44 | 35.0 | 1122 | 3 | 34 | 4 | 1271 | 108 |
| wAus | 1.16 | 34.0 | 1040 | 3 | 34 | 4 | 1124 | 43 |
| wOb | 1.12 | 33.8 | 952 | 4 | 33 | 3 | 1118 | 126 |
| wLcla | 1.15 | 34.1 | 933 | 3 | 35 | 3 | 1183 | 209 |
| wVitB | 1.11 | 34.0 | 900 | 4 | 36 | 4 | 1074 | 130 |
| wDacB | 1.50 | 34.0 | 1246 | 3 | 40 | 4 | 1592 | 299 |
| wTpre | 1.13 | 33.9 | 827 | 3 | 35 | 4 | 1106 | 237 |
| wStri | 1.79 | 33.9 | 1447 | 3 | 34 | 4 | 1725 | 237 |
| wDi | 1.24 | 34.3 | 1035 | 3 | 34 | 4 | 1215 | 139 |
| China 1 | 1.31 | 35.1 | 977 | 3 | 37 | 4 | 1217 | 196 |
| wAlbB | 1.48 | 34.4 | 1206 | 3 | 34 | 4 | 1427 | 180 |
| wLug | 1.54 | 33.6 | 1259 | 3 | 34 | 4 | 1501 | 201 |
| wMau | 1.27 | 34.0 | 1047 | 3 | 34 | 4 | 1219 | 131 |
| wNo | 1.30 | 34.0 | 1066 | 3 | 34 | 4 | 1240 | 133 |
| wCon | 2.11 | 34.7 | 2166 | 3 | 34 | 1 | 2393 | 189 |
| wVulC | 1.66 | 34.5 | 1301 | 3 | 35 | 4 | 1672 | 329 |
| wOo | 0.96 | 32.1 | 639 | 3 | 34 | 4 | 753 | 73 |
| wOv | 0.96 | 32.1 | 649 | 3 | 34 | 4 | 763 | 73 |
| wCle | 1.25 | 36.3 | 1012 | 3 | 34 | 4 | 1263 | 210 |
| wBm | 1.08 | 34.2 | 805 | 3 | 34 | 4 | 1024 | 178 |
| wWb | 1.06 | 34.3 | 961 | 3 | 33 | - | 997 | 1 |
| wFol | 1.80 | 34.4 | 1509 | 3 | 35 | 4 | 1649 | 98 |

**Note:** - represents no annotation data.
